# Performing the hand laterality judgement task does not necessarily require motor imagery

**DOI:** 10.1038/s41598-020-61937-9

**Published:** 2020-03-20

**Authors:** Akira Mibu, Shigeyuki Kan, Tomohiko Nishigami, Yuji Fujino, Masahiko Shibata

**Affiliations:** 1 Department of Physical Therapy, Konan Women’s University, 6-2-23 Morikita-machi, Higashinada-ku, Kobe, Hyogo 658-0001 Japan; 2 Department of Anesthesiology and Intensive Care Medicine, Osaka University Graduate School of Medicine, 2-2 Yamadaoka, Suita, Osaka 565-0871 Japan; 3 Department of Physical Therapy, Prefectural University of Hiroshima, 1-1 Gakuen-cho, Mihara, Hiroshima 723-0053 Japan; 4 Faculty of Health Science, Naragakuen University, 3-15-1 Nakatomigaoka, Nara, Nara 631-8524 Japan

**Keywords:** Motor control, Human behaviour

## Abstract

When people judge the laterality of rotated hand images, that is they perform the laterality judgement task (LJT), they are thought to use motor imagery. However, recent studies have suggested that its completion does not necessarily require the use of motor imagery. In this study, we investigated whether and how many people preferentially use motor imagery to perform the LJT in 37 healthy adults. We assessed the presence of behavioural features associated with motor imagery at the individual level, namely, the linear angle–response time (RT) relationship and the biomechanical constraints effect in the LJT and in the same-different judgement task (SDJT), in which people are not thought to use motor imagery. We found that at most 50% of participants showed both behavioural features in the palmar view condition of the LJT. Moreover, this proportion did not differ from that in the dorsal view condition of the LJT or that in both view conditions of the SDJT. These results demonstrate that a motor imagery–based strategy is not universally and specifically used to perform the LJT. Therefore, previous results of the LJT, in particular, regarding the biomechanical constraints effect, should be reinterpreted in light of our findings.

## Introduction

Motor imagery is a mental process by which people rehearse or simulate an action in their mind without actually performing the movement^1–3^. Because motor imagery shares control mechanisms with actual movement^4,5^, the ability to perform motor imagery is considered to reflect the ability to perform an actual movement^6^. Based on this assumption, motor imagery is gathering much attention as a tool in sports training and rehabilitation^6–8^.

The laterality judgement task (LJT) requires participants to judge the laterality of presented hand images, and is widely used to measure motor imagery ability in healthy people^9^. Furthermore, the LJT is becoming more popular in clinical practice to evaluate and restore motor imagery ability in patients with movement dysfunction^8,10–12^ and with chronic pain^13^. To perform the task, it is assumed that people use motor imagery. Specifically, it is believed that people simulate the kinematic properties of the physical action of their own hand mentally from an initial resting position (usually an upright position) to the position of a presented hand image^14,15^. This cognitive process is referred to as a motor imagery–based strategy. However, recent studies have suggested that strategies other than motor imagery are used to perform the LJT^16,17^. For example, some people are thought to use the visual mental rotation of the presented hand image (a visual imagery-based strategy)^16,18^ or simple comparisons of figure shapes without rotation^19^. Therefore, it is still an open question whether people execute motor imagery during the LJT.

According to previous studies, two behavioural features have been regarded as indicators of using motor imagery to perform the LJT. One is a linear relationship between rotation angles and response times (RTs), which is regarded as a behavioural feature of performing motor mental rotation of one’s own hand^20,21^, as well as a visual mental rotation of a picture of a hand^22^. The other is the biomechanical constraints effect, a phenomenon in which RTs for hand pictures rotated laterally (fingers pointing away from the body) are larger than those rotated medially in the LJT. This phenomenon is regarded as a behavioural feature of performing motor imagery of hands because the RT increase for lateral rotation is thought to stem from the fact that it is more difficult to rotate hands laterally than to rotate them medially^14,15^. The presence of these two features has been demonstrated independently at the group level^14–16,18,20–25^. However, the presence of the biomechanical constraints effect at the individual level has not been investigated systematically. Moreover, the coexistence of these two features has not been examined at either the group or individual levels; an assessment of the presence of these features is necessary to determine whether people actually perform mental rotation of their hands to perform the LJT. Indeed, given that the LJT is used as a tool for sports training and rehabilitation, it is essential to establish whether each individual uses a motor imagery–based strategy to perform this task.

In this study, therefore, we investigated whether and how many people use a motor imagery–based strategy to perform the LJT by assessing the two behavioural features in each participant. If motor imagery is necessary to perform the LJT, almost all participants should show the presence of both a linear angle–RT relationship and the biomechanical constraints effect. Additionally, we tested the conventional view of preferred strategies for hand mental rotation tasks. It is currently thought that motor imagery is used in the palmar view condition (palm-side hand images are presented) of the LJT whereas visual imagery is used in the LJT dorsal view condition and in both palmar and dorsal view conditions of the same-different judgement task (SDJT), in which people judge whether the laterality of two hand images shown concurrently is the same^20,21^. If this conventional view was true, the linear angle–RT relationship should be observable under all conditions, whereas the biomechanical constraints effect should be evident in only the palmar view condition of the LJT. That is, the number of participants showing both behavioural features in parallel should differ significantly between the palmar view condition of the LJT and other conditions. Thus, we compared the numbers of participants showing both behavioural features between view conditions (dorsal vs. palmar view) and between tasks (LJT vs. SDJT).

## Results

### The relationship between rotation angle and response time at the group level

**Figure 1:**
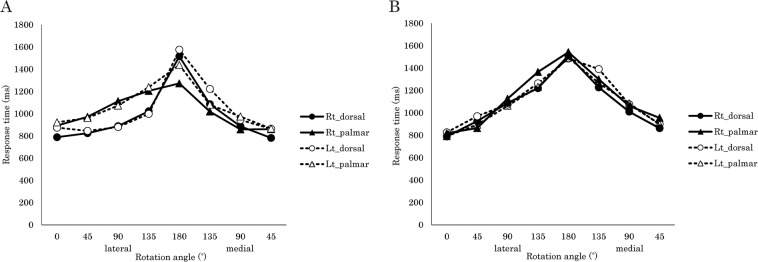
Relationship between rotation angle and response time at the group level. Mean response time in the (**A**) laterality judgement task, and (**B**) same–different judgement task. Rt_dorsal: right hand–dorsal view; Rt_palmar: right hand–palmar view; Lt_dorsal: left hand–dorsal view; Lt_palmar: left hand–palmar view; lateral: lateral rotation; medial: medial rotation.

Figure 1 shows the group mean RTs at each rotation angle for the LJT and the SDJT. In the LJT, participants were requested to identify the laterality of a displayed hand image. For the SDJT, they were requested to identify whether the laterality of simultaneously displayed two hand images were same (see Methods for details). In each stimulus condition of both tasks, simple regression analyses revealed that all slopes of regression lines between rotation angles of displayed hand images and RTs were significantly positive (all, p < 0.001), which indicates that RTs linearly increased from 0° to 180°. Group mean RT values and all simple regression analyses results are shown in Supplementary Tables 1 and 2, respectively.

### The presence of the biomechanical constraints effect at the group level

**Figure 2:**
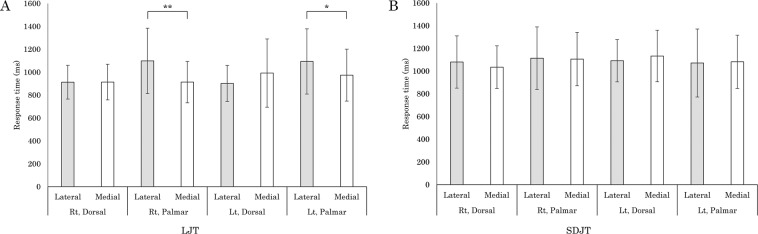
Comparisons of mean response times between medial and lateral rotation at the group level. Mean response times for medial and lateral rotation in each stimulus condition in the (**A**) laterality judgement task and (**B**) same–different judgement task. Error bars represent the standard deviations. *p < 0.01; **p < 0.001. LJT: laterality judgement task; SDJT: same–different judgement task; lateral: lateral rotation; medial: medial rotation; Rt: right hand; Lt: left hand; Dorsal: dorsal view; Palmar: palmar view.

To test the presence of the biomechanical constraints effect at the group level, we compared group mean RTs between medial and lateral rotation in each stimulus condition of the LJT and SDJT using paired t-tests (Fig. 2). For the LJT, mean RTs of the lateral rotation were significantly longer than those of the medial rotation in the right hand–palmar view condition (lateral rotation: 1098.5 ± 285.6 ms; medial rotation: 913.5 ± 180.3 ms; t(26) = 4.74, p < 0.001) and the left hand–palmar view condition (lateral rotation: 1093.9 ± 285.1 ms; medial rotation: 974.0 ± 226.7 ms; t(26) = 3.65, p < 0.001). In the SDJT, no significant RT differences between lateral and medial rotation were found in any stimulus condition (all, p > 0.08). Paired t-test results are shown in Supplementary Table 3.

### The presence of the biomechanical constraints effect and the linear angle–RT relationship at the individual level

**Figure 3:**
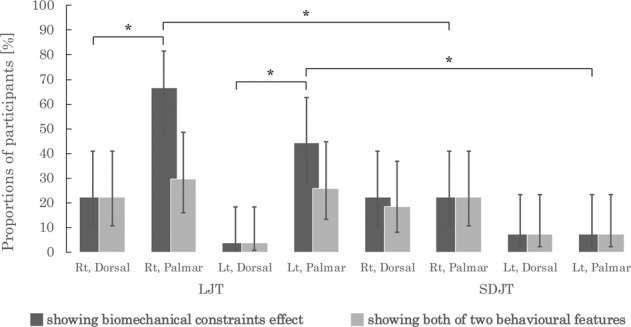
Proportions of participants showing the biomechanical constraints effect and showing both behavioural features (significance level in the individual level analysis: p < 0.05). Error bars represent 95% confidence intervals. *p < 0.00625 (corrected for multiple comparisons using the Bonferroni method). Rt: right hand; Lt: left hand; Dorsal: dorsal view; Palmar: palmar view; LJT: laterality judgement task; SDJT: same-different judgement task.

To investigate how many people showed the biomechanical constraints effect and the linear angle–RT relationship, we also tested them at the individual level (in each test, the threshold was set at p < 0.05). We then compared the number of participants who showed (1) the biomechanical constraints effect, (2) the linear angle–RT relationship, (3) both and (4) neither of them between views (dorsal vs. palmar) and between tasks (LJT vs. SDJT). Table 1 presents the proportions of the participants that showed the biomechanical constraints effect, the linear angle–RT relationship, both, and neither. As shown in Table 1 and Fig. 3, a total of 44% to 67% of participants showed the biomechanical constraints effect in the palmar view condition of the LJT, while only up to 22% of participants showed this effect in the dorsal view condition of the LJT and in all conditions of the SDJT.

**Table 1:** Proportions of participants showing the biomechanical constraints effect, the linear angle-RT relationship, both effects, and neither.

Task	Alpha level in the individual level analysis	Condition	Proportion and 95% CI of the participants showing:			
			Biomechanical constraints effect	Linear angle-RT relationship	Both of these features	None of these features
LJT^a^	0.05	Rt, Dorsal	22.2 (10.6–40.8)	92.6 (76.6–97.9)	22.2 (10.6–40.8)	7.4 (2.1–23.4)
		Rt, Palmar	66.7 (47.8–81.4)	51.9 (34.0–69.3)	29.6 (15.9–48.5)	11.1 (3.9–28.1)
		Lt, Dorsal	3.7 (0.7–18.3)	88.9 (71.9–96.1)	3.7 (0.7–18.3)	11.1 (3.9–28.1)
		Lt, Palmar	44.4 (27.6–62.7)	74.1 (55.3–86.8)	25.9 (13.2–44.7)	7.4 (2.1–23.4)
	0.2	Rt, Dorsal	29.6 (15.9–48.5)	96.3 (81.7–99.3)	29.6 (15.9–48.5)	3.7 (0.7–1.8)
		Rt, Palmar	70.4 (51.5–84.1)	70.4 (51.5–84.1)	48.1 (30.7–66.0)	7.4 (2.1–23.4)
		Lt, Dorsal	22.2 (10.6–40.8)	92.6 (76.6–97.9)	22.2 (10.6–40.8)	7.4 (2.1–23.4)
		Lt, Palmar	63.0 (44.2–78.5)	74.1 (55.3–86.8)	40.7 (24.5–59.3)	3.7 (0.7–1.8)
SDJT^a^	0.05	Rt, Dorsal	22.2 (10.6–40.8)	96.3 (81.7–99.3)	18.5 (8.2–36.7)	0
		Rt, Palmar	22.2 (10.6–40.8)	96.3 (81.7–99.3)	22.2 (10.6–40.8)	3.7 (0.7–18.3)
		Lt, Dorsal	7.4 (2.1–23.4)	92.6 (76.6–97.9)	7.4 (2.1–23.4)	7.4 (2.1–23.4)
		Lt, Palmar	7.4 (2.1–23.4)	92.6 (76.6–97.9)	7.4 (2.1–23.4)	7.4 (2.1–23.4)
	0.2	Rt, Dorsal	51.9 (34.0–69.3)	96.3 (81.7–99.3)	48.1 (30.7–66.0)	0
		Rt, Palmar	29.6 (15.9–48.5)	100	29.6 (15.9–48.5)	0
		Lt, Dorsal	18.5 (8.2–36.7)	100	18.5 (8.2–36.7)	0
		Lt, Palmar	18.5 (8.2–36.7)	92.6 (76.6–97.9)	18.5 (8.2–36.7)	7.4(2.1–23.4)

^a^n = 27. LJT: laterality judgement task; SDJT: same-different judgement task; Rt: right hand; Lt: left hand; Dorsal: dorsal view; Palmar: palmar view; CI: confident interval.

In contrast to the biomechanical constraints effect, at least 52% of participants showed the linear angle–RT relationship in all stimulus conditions of both tasks except for the right hand–palmar view condition of the LJT. Moreover, at most 30% of participants showed both the biomechanical constraints effect and the linear angle-RT relationship in the LJT, except for the left hand–dorsal view condition (Fig. 3).

For both hands, a larger proportion of participants showed the biomechanical constraints effect in the palmar view condition than in the dorsal view condition of the LJT (both hands, p < 0.003, Table 2 and Fig. 3). In contrast, between-view comparisons in the proportions of the participants showing both behavioural features failed to reveal any significant differences in either the right- or left-hand condition of each task (all, p > 0.03, Table 2). All statistical values of between-view comparisons are shown in Supplementary Table 4.

**Table 2:** Results of between-view comparisons in the LJT and SDJT.

Task	Alpha level in the individual level analysis	Hand	Participants showing:			
			Biomechanical constraints effect	Linear angle–RT relationship	Both of these features	None of these features
LJT	0.05	Right	0.003*	<0.001*	0.564	0.560
		Left	0.002*	0.157	0.034	0.655
	0.2	Right	0.005*	0.020	0.132	0.564
		Left	0.008	0.059	0.166	0.564
SDJT	0.05	Right	1.00	1.00	0.706	0.317
		Left	1.00	1.00	1.00	1.00
	0.2	Right	0.033	0.317	0.059	NA
		Left	1.00	0.157	1.00	0.157

Each value is a p-value. An asterisk (*) indicates a significant difference between the dorsal and palmar view (threshold: p = 0.00625, corrected for multiple comparisons using the Bonferroni method). LJT: laterality judgement task; SDJT: same-different judgement task; RT: response time; NA: not applicable.

In the palmar view condition, the proportion of participants showing the biomechanical constraints effect in the LJT were bigger than those in the SDJT (both hands, p < 0.004, Table 3 and Fig. 3). However, in participants showing both behavioural features, there were no significant between-task differences in the number of participants in any of the hand × view conditions (all, p > 0.06, Table 3). Between-task comparison results are shown in Supplementary Table 6.

**Table 3:** Results of between-task comparisons in each condition.

Hand	Alpha level in the individual level analysis	View	Participants showing:			
			Biomechanical constraints effect	Linear angle–RT relationship	Both of these features	None of these features
Right	0.05	Dorsal	1.00	0.564	0.739	0.157
		Palmar	0.003*	<0.001*	0.527	0.317
	0.2	Dorsal	0.083	1.000	0.166	0.317
		Palmar	0.012	0.005*	0.197	0.157
Left	0.05	Dorsal	0.564	0.655	0.564	0.655
		Palmar	0.004*	0.059	0.059	1.00
	0.2	Dorsal	0.739	0.157	0.739	0.157
		Palmar	0.005*	0.059	0.109	0.317

Each value is a p-value. An asterisk (*) indicates a significant difference between the laterality judgement task and same-different judgement task (threshold: p = 0.00625, corrected for multiple comparisons using the Bonferroni method). RT: response time; NA: not applicable.

As mentioned above, we set the thresholds for individual level analysis (regression analysis and t-test, each) at p < 0.05. This threshold, however, may be too strict to find both the linear angle–RT relationship and the biomechanical constraints effect in a single person. Consequently, we possibly underestimated the number of people showing both features in parallel in the LJT. To test this possibility, we applied a more lenient threshold (p < 0.2) to the individual level analysis and conducted between-view and between-task comparison again (detailed results of between-view and between-task comparisons are shown in Supplementary Tables 5 and 7, respectively). Even at the lenient threshold, at most 48.1% of participants showed both features in the LJT (Table 1). Furthermore, any comparisons in number of participants showing both behavioural features did not reach the significance level (p = 0.00625; Tables 2 and 3, Supplementary Fig. 1). This was the same result when the strict significance level (p < 0.05) was applied to the individual level analysis.

## Discussion

It is not yet clear whether a motor imagery–based strategy is universally and specifically adopted to perform the LJT. We therefore assessed the presence of behavioural features of performing motor imagery (the biomechanical constraints effect and the linear angle–RT relationship) in individual participants. According to widely accepted views, most individuals would be expected to show both the biomechanical constraints effect and the linear angle–RT relationship. However, even at the lenient threshold (p < 0.2), we found that the biomechanical constraints effect was not observed in about 30% to 37% of our participants in the palmar view condition of the LJT. Moreover, this effect was not always concomitant of the linear angle–RT relationship. Specifically, 52% to 60% of participants did not show both of the two behavioural features in this view condition. These findings indicate that many individuals do not use the motor imagery–based strategy to perform the LJT, and that the biomechanical constraints effect alone does not necessarily reflect motor imagery. Furthermore, between-view (palmar vs. dorsal) and between-task (LJT vs. SDJT) comparisons showed that there were no differences in the number of participants showing both behavioural features of the motor imagery, which indicates that motor imagery is not specifically used in the palmar view condition of the LJT. Considering these findings, previous results of the LJT, in particular, those regarding the biomechanical constraints effect, should be reinterpreted in light of our findings, and the use of this task as a tool for measuring motor imagery ability, particularly in clinical practice, should be called into question.

In the present study, the mean group RTs monotonically increased from 0° to 180° in each stimulus condition, and the biomechanical constraints effect was observed only in the palmar view condition of the LJT. These two results were consistent with previous studies regarding the linear angle-RT relationship^14–16,18,20–24^ and the biomechanical constraints effect^14–16,18,20,21,23–25^. Although there were several procedural differences between the present study and previous ones, these did not affect participants’ behaviour, particularly strategy preference for hand mental rotation tasks.

In this study, even at the lenient threshold, at least 30% of participants did not show the biomechanical constraints effect in the palmar view condition of the LJT. Moreover, only 48% of participants showed the biomechanical constraints effect and the linear angle–RT relationship in parallel. These results are surprising, because numerous studies have demonstrated the presence of the biomechanical constraints effect in this condition at the group level^14–16,18,20,21,23–25^, and it is widely accepted that the motor imagery–based strategy is universally used in this condition to perform this task. However, these results are in line with a few recent studies that have reported that individuals do not perform motor imagery during the LJT. For example, Berneiser *et al*. found that the biomechanical constraints effect was observed only after training with the LJT in healthy individuals^16^. There is one possible explanation for this result, that the presence of the biomechanical constraints effect corresponds to the performance level in this task. However, the average RT in the present study was comparable with that in their study and individual RTs in this study did not differ among participants regardless of the presence of the biomechanical constraints effect. Therefore, that explanation is not applicable to our result. In addition to results from Berneiser *et al*., Sekiyama also reported that the biomechanical constraints effect was not observed in the group showing a peak of RT profile at 180°^14^. Furthermore, Ferron and Tremblay reported that motor evoked potentials (MEP) were not enhanced while participants performed the LJT^17^. Given that the primary motor cortex is involved in both motor imagery and motor execution^26^, this result implies that participants did not use motor imagery to perform the LJT. Although several studies reported the significant increases in MEP amplitude during motor imagery tasks^27^ and the LJT^28^, the inconsistencies in previous studies suggest that participants may use different strategies to perform the LJT depending on experimental settings. Our results demonstrate that the motor imagery–based strategy is not used consistently to perform the LJT on an individual level, which previous studies have suggested at the group level. The results also indicate that, contrary to previous assumptions, at least one third of the people do not use a motor imagery–based strategy to perform the LJT.

In addition to the necessity of using motor imagery to perform the LJT, our result, in particular, the presence of the biomechanical constraints effect was not in parallel with that of the linear angle–RT relationship in many participants, raises an intriguing question about the interpretation of the biomechanical constraints effect. That is, RT difference between lateral and medial rotation would not reflect performing the motor imagery when RTs do not increase as a function of rotation angle. The linear angle–RT relationship is considered as an indicator of performing motor^21,22^ as well as visual mental rotation^20^. If the biomechanical constraints effect is indicative of motor imagery (that is, motor mental rotation) during the LJT, this effect should be observed along with the linear angle–RT relationship. However, even in the palmar view condition of the LJT, in which the largest number of participants showed the biomechanical constraints effect, only 40% to 48% of participants showed both behavioural features in parallel. Furthermore, while about 63% to 70% of participants showed the biomechanical constraints effect, about 35% of them did not show the linear angle–RT relationship. These results clearly indicate that the biomechanical constraints effect alone does not necessarily indicate the use of hand motor imagery for the LJT and support recent reports that the biomechanical constraints effect reflects cognitive processes other than motor imagery. For example, Vannuscorps *et al*. investigated LJT performance in a patient with congenital absence of the upper limbs^29^. Although the patient was not able to perform motor imagery as well as motor execution of hands, he showed the biomechanical constraints effect in the LJT. Meng *et al*. investigated brain regions related to the biomechanical constraints effect to examine the notion that the biomechanical constraints effect depends on performing motor imagery^30^. The biomechanical constraints effect is thought to reflect the fact that lateral rotation of the hands is more difficult than medial rotation, and so brain regions related to this effect should show stronger activation during the lateral rotation than the medial rotation. Although the superior parietal lobule showed this expected activation pattern, motor areas did not. Considering our results, the participants in these previous studies may have been individuals who only showed the biomechanical constraints effect in the LJT. Although we cannot determine what cognitive processes the biomechanical constraints effect might reflect when it was not in parallel with the presence of the linear angle–RT relationship, our results emphasize the need for further research into what the biomechanical constraints effect reflects when the linear angle-RT relationship is not present in parallel with this effect.

Between-view (dorsal vs. palmar) and between-task (LJT vs. SDJT) comparisons in the number of participants who showed both behavioural features associated with motor imagery raise questions about the assumption of preferred strategies for the LJT and SDJT, in particular, whether the motor imagery–based strategy is preferentially adopted in the palmar view condition of the LJT. Previous studies^21,22^ have reported that a linear angle–RT relationship was observed in the dorsal view condition of the LJT as well as in all conditions of the SDJT, but the biomechanical constraints effect was not. Therefore, in such conditions of such tasks, the motor imagery–based strategy is not thought to be used. However, in the present study, 8% to 25% of participants showed both behavioural features in the dorsal view condition of the LJT and all conditions of the SDJT, even when strict significance level (p < 0.05) was applied to the individual level analysis. Furthermore, the numbers of such participants in these conditions were not significantly different compared with those in the palmar view condition of the LJT. This indicates that up to 25% of people use the motor imagery–based strategy in hand mental rotation tasks, regardless of stimulus condition and task type.

Previous studies have argued that motor imagery is performed implicitly in the LJT^31–35^. Parsons reported that several participants showed the biomechanical constraints effect in the LJT without being aware of performing motor imagery^31^. Their group also demonstrated that brain regions related to somatosensory and motor processing were activated during the LJT in a modality specific manner by using positron emission tomography^32^. These results suggest that motor imagery is conducted unconsciously during the LJT. Moreover, several previous studies reported brain activity in motor-related regions during not only the LJT^33^ but also the SDJT^34,35^. However, because this issue was not our interest, we did not directly address this. Nevertheless, it is true that some participants showed both behavioural features associated with motor imagery in the LJT and SDJT in this study. We did not offer any suggestions about strategies for hand mental rotation tasks. Therefore, if participants in the present study were not aware of performing motor imagery, our results possibly support the assumption that “implicit” motor imagery contributes to complete hand mental rotation tasks.

The LJT is becoming popular in clinical practice to evaluate and restore motor imagery ability in patients with movement dysfunction^8,10–12^ and with chronic pain^13^. Previous studies investigating the ability of hand laterality judgement in patients with stroke^10,11^, Parkinson’s disease^12^, and complex regional pain syndrome (CRPS)^13^ have reported a worse performance of the LJT (i.e., RTs and accuracy) on the affected side compared with the healthy side or to healthy people. These studies concluded that this deterioration resulted from the dysfunction of motor execution and motor imagery for the affected hand. However, our results showed that more than 70% of people do not perform motor imagery during the LJT, and that the biomechanical constraints effect alone does not indicate the use of hand motor imagery. Therefore, deterioration of LJT performance in such patients may not be related to deterioration of the motor imagery ability. Because individual RTs reflect cognitive ability related to a strategy adopted to complete a task, RTs cannot reflect their motor imagery ability in participants who do not use motor imagery during the LJT. Our findings also could explain the low effectiveness of the LJT on patients. A previous meta-analysis for the effectiveness of the LJT in patients with CRPS reported that the effect of the LJT on pain relief was positive, but not significantly different to that of usual care^36^. Considering our findings, the LJT may be less effective for CRPS patients using strategies other than motor imagery in this task. Conversely, the LJT may be effective for patients who use motor imagery to complete this task. Therefore, responders to the LJT in such patients could be predicted by assessing whether motor imagery is used to complete the LJT, that is, whether the biomechanical constraints effect and the linear angle–RT relationship coexist in the LJT.

The most significant limitation of this study is that we cannot conclude what the biomechanical constraints effect reflects. Furthermore, although our behavioural results indicate that many people do not use the motor imagery–based strategy for the LJT, we were not able to specify strategies that they did use. Besides RT profiles, verbal reports about response strategies^37^, eye movement patterns^38^, and brain activity during the LJT^39–41^ at the individual level would be helpful measures to address these issues.

Another limitation is that we tested strategies for the LJT and SDJT only once in each participant. That is, we did not consider the possibility that strategies for hand mental rotation tasks may change through training. Although multiple factors are assumed to be involved in training–induced performance improvement^42^, one possible cause is thought to be a change of strategy^43,44^. Previous studies have, in fact, suggested strategy change after training in a working memory task^45^ and a mental rotation task of 3D objects^46^. Furthermore, as mentioned above, Berneiser *et al*. showed that training for the LJT led to performance improvement and a strategy change from a visual imagery–based strategy to a motor imagery–based strategy in healthy people^16^. In light of these findings, further studies are needed to generalize our findings.

In conclusion, our findings challenge the widely-accepted assumption that motor imagery-based strategy is universally used to perform the LJT. Our findings also raise the question of what the biomechanical constraints effect reflects when the linear angle–RT relationship is not present in parallel with this effect. Therefore, previous results of the LJT should be reinterpreted in light of our findings, and use of the LJT as a tool for measuring motor imagery ability, particularly in clinical practice, should be reconsidered.

## Methods

### Participants

Thirty-seven healthy adults (18 female; mean age ± SD: 21.2 ± 1.1 years old) participated in this study. All participants were right-handed as assessed by Japanese version of the FLANDERS handedness questionnaire^47,48^ (mean score ± SD: 9.5 ± 1.1) and they had normal or corrected-to-normal vision. All participants provided informed consent before their participation. This study was approved by the ethical committee of Osaka University Hospital and followed the Declaration of Helsinki.

### Mental rotation task and visual stimuli

We used two types of hand mental rotation tasks, as follows: the laterality judgement task (LJT) and the same–different judgement task (SDJT). In the LJT, participants judged the laterality of a rotated hand image. In the SDJT, they judged whether the laterality of two simultaneously presented hand images were the same or not. During the SDJT, a reference stimulus was always presented on the left-hand side in the upright position, and a rotated test stimulus was presented on the right. For both tasks, the test stimuli were presented at eight different rotation angles, as follows: 0° (upright position), 45°, 90°, 135°, 180°, 225°, 270°, and 315° in a clockwise direction. Therefore, a set of 32 hand images (2 hands [left/right hand] × 2 views [dorsal/palmar] × 8 rotation angles) was used in each task.

### Experimental setup

Participants performed the hand mental rotation tasks in a quiet room under normal lighting conditions. They sat comfortably with their hands resting on their thighs, at a distance of about 50 cm from a computer screen. Participants responded using two foot- switches that were positioned on the floor within a comfortable reaching distance. Stimulus presentation was controlled and participants’ responses were acquired using Presentation (Neurobehavioral systems, Albany, USA).

### Procedure

The LJT and SDJT were conducted on the same day. The order of the tasks was counterbalanced across participants. Each task consisted of one practice block and four experimental blocks, and each block contained 96 trials. In each experimental block, the set of 32 hand images was presented three times in a random order; thus, each hand image was presented 12 times throughout all four experimental blocks. The stimuli (rotated hand images) appeared and remained visible on the screen until participants gave a response by pressing the foot switch. Participants were asked to respond as quickly and accurately as possible and not to look at or move their hands when judging. Participants could take a break between blocks, and they decided the length of breaks.

### Data analysis

We computed mean response times (RTs) in each combination among stimulus conditions of both tasks at the individual level, and only included correct response trials. We also excluded trials for which RTs were more than and less than 2 SDs from the calculation of mean RTs in each participant. Participants with a high error rate (more than 50%) for any of the stimulus conditions in either the LJT or the SDJT were excluded from the analyses at the group and individual level. In the result section, RTs are presented as the mean ± SD.

At the group level, to examine whether RTs linearly increase with rotation angles, we tested the linearity of the angle–RT relationship using a simple regression analysis for each stimulus condition (hand [left/right] × view [dorsal/palmar view] × direction of rotation [medial/lateral rotation]). If the slope of a regression line between 0° to 180° was significantly positive, we regarded RTs to be monotonically increased with rotation angles.

To test the presence of the biomechanical constraints effect at the group level, we compared mean RTs between medial and lateral rotations in each stimulus condition using a one-tailed paired t-test in the LJT and SDJT. For the left-hand images, the medial rotation corresponded to 45°, 90° and 135°, and the lateral rotation corresponded to 315°, 270° and 225°. Conversely, for the right-hand images, the medial rotation corresponded to 315°, 270° and 225°, and the lateral rotation corresponded to 45°, 90° and 135°. In these cases, 315°, 270° and 225° were respectively regarded as 45°, 90° and 135° from 0° in a counter-clockwise direction^14,49^. We considered the biomechanical constraints effect to be present if the mean RT of lateral rotation was significantly longer than that of medial rotation.

To test the presence of the biomechanical constraints effect at the individual level, we compared mean RTs between medial and lateral rotation in each participant for each stimulus condition using a one-tailed two-sample t-test. To test the presence of the linear angle–RT relationship at the individual level, we also conducted a simple regression analysis for individual RT data. Full results of these individual-level analyses are shown in Supplementary Tables 6 and 7. Based on these results, we calculated the proportion of participants showing the biomechanical constraints effect, the linear angle–RT relationship, both, and neither, respectively. Furthermore, we examined between-view and between-task differences in these proportions using a McNemar test.

Statistical tests were conducted using the data analysis software JMP (SAS institute Inc., Cary, NC, USA). The significance level was set at p < 0.05 for all statistical tests. Additionally, we also applied a more lenient significance level (p < 0.2) to simple regression analysis and t-test at the individual level. To control for type I errors in multiple comparisons, a Bonferroni correction was applied to the between-view and between-task comparisons by the McNemar test. In these comparisons, a series of eight tests was performed for each task or hand condition. Therefore, the significance level was p < 0.00625 (= 0.05/8) in these comparisons.

## Supplementary information

Supplementary figures and Tables.

## Data Availability

The datasets generated and analysed during the current study are available from the corresponding author on reasonable request.
