# Supplementary material for: Performing the hand laterality judgement task does not necessarily require motor imagery
Source: Sci Rep. 2020 Mar 20;10:5155. doi: 10.1038/s41598-020-61937-9 (PMC7083854; doi:10.1038/s41598-020-61937-9)
Supplement: Supplementary file 1 — Supplementary figures and Tables. [file 41598_2020_61937_MOESM1_ESM.pdf]

Title:

Performing the hand laterality judgement task does not necessarily require motor imagery

Author names and affiliations:

Akira Mibu[1,2], Shigeyuki Kan[2*], Tomohiko Nishigami[1], Yuji Fujino[2], Masahiko Shibata[2,3]

[1]Department of Physical Therapy Konan Women's University

6-2-23 Morikita-machi, Higashinada-ku, Kobe, Hyogo 658-0001, Japan

[2]Department of Anesthesiology and Intensive Care Medicine, Osaka University Graduate School of Medicine

2-2 Yamadaoka, Suita, Osaka 565-0871, Japan

[3]Faculty of Health Science, Naragakuen University

3-15-1 Nakatomigaoka, Nara, Nara 631-8524, Japan

*Corresponding author:

Shigeyuki Kan

Tel: +81-6-6879-3133

Fax: +81-6-6879-3139

E-mail: skan@anes.med.osaka-u.ac.jp

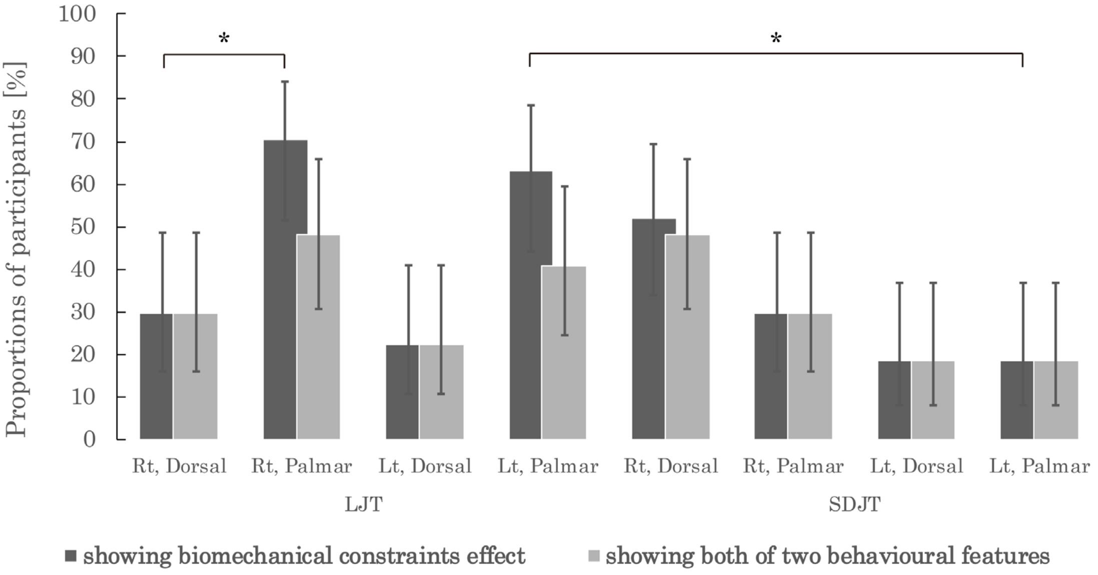

Supplementary figure 1. Proportions of participants showing the biomechanical constraints effect and showing both behavioural features (significance level in the individual level analysis: $p < .2$). Error bars represent 95% confidence intervals. *$p < .00625$ (corrected for multiple comparisons using the Bonferroni method). Rt: Right; Lt: Left; Dorsal: dorsal view; Palmar: palmar view; LJT: laterality judgement task; SDJT: same-different judgement task.

Supplementary table 1. Group mean response time and standard deviation

	Condition			0°		45°		90°		135°		180°	
Task	Hand	View	Rotation	Mean	SD	Mean	SD	Mean	SD	Mean	SD	Mean	SD
LJT	Right	Dorsal	Lateral	789.0	103.4	824.6	123.4	888.9	146.2	1022.6	222.8	1515.4	528.6
			Medial	789.0	103.4	782.1	144.2	886.9	157.8	1086.8	287.5	1515.4	528.6
		Palmar	Lateral	893.0	176.4	972.2	242.2	1112.9	398.6	1205.1	327.2	1272.6	345.2
			Medial	893.0	176.4	860.6	162.2	858.6	152.5	1019.3	275.2	1272.6	345.2
	Left	Dorsal	Lateral	875.0	219.4	845.7	156.4	879.9	142.5	998.6	212.4	1575.9	682.3
			Medial	875.0	219.4	864.4	149.4	943.5	190.1	1222.9	639.4	1575.9	682.3
		Palmar	Lateral	922.7	189.3	961.5	215.3	1073.1	314.7	1239	420.4	1438.5	386.0
			Medial	922.7	189.3	866.6	146.0	974.4	255.9	1081.6	359.4	1438.5	386.0
SDJT	Right	Dorsal	Lateral	789.0	154.1	926.3	173.5	1088.5	258.1	1220.5	280.9	1511.8	424.9
			Medial	789.0	154.1	863.0	169.7	1011.1	195.5	1227.2	232.9	1511.8	424.9
		Palmar	Lateral	817.9	131.5	864.4	162.3	1126.7	445.7	1365.9	307.2	1542.3	481.5
			Medial	817.9	131.5	955.7	195.0	1061.9	223.4	1299.6	333.7	1542.3	481.5
	Left	Dorsal	Lateral	826.2	162.2	970.5	161.9	1074.9	216.3	1263.9	308.4	1485.1	424.3
			Medial	826.2	162.2	900.4	181.6	1076.9	241.7	1391.1	291.3	1485.1	424.3
		Palmar	Lateral	792.3	146.4	894.5	199.7	1064.1	273.0	1231.7	387.5	1494.1	347.7
			Medial	792.3	146.4	898.3	173.8	1084.9	265.5	1266.3	327.3	1494.1	347.7

Note. Between medial and lateral rotation, response times are the same at 0° and 180, respectively. LJT = Laterality Judgment Task. SDJT = Same-Different Judgment Task. SD = standard error.

Supplementary table 2. Results of simple regression anlysis (statistical test for significance of coefficient)

	Condition			Coefficient			
Task	Hand	View	Direction	Beta	SE	t	p
LJT	Right	Dorsal	Lateral	3.67	0.41	9.04	< .001
			Medial	3.91	0.42	9.34	< .001
		Palmar	Lateral	2.20	0.41	5.34	< .001
			Medial	2.04	0.34	6.06	< .001
	Left	Dorsal	Lateral	3.45	0.52	6.69	< .001
			Medial	3.91	0.61	6.40	< .001
		Palmar	Lateral	2.91	0.43	6.76	< .001
			Medial	2.77	0.40	6.87	< .001
SDJT	Right	Dorsal	Lateral	3.87	0.37	10.37	< .001
			Medial	4.02	0.35	11.48	< .001
		Palmar	Lateral	4.33	0.46	9.51	< .001
			Medial	3.98	0.40	9.84	< .001
	Left	Dorsal	Lateral	3.58	0.37	9.73	< .001
			Medial	4.02	0.38	10.70	< .001
		Palmar	Lateral	3.86	0.39	10.04	< .001
			Medial	3.94	0.36	11.05	< .001

Note. LJT = Laterality Judgment Task. SDJT = Same-Different Judgment Task. SE = standard error. df = degree of freedom

Supplaementary table 3. Comparisons of RTs between medial and lateral rotation at the groupl level

	Condition		Response time		Lateral > Medial		
			Lateral	Medial			
Task	Hand	Side	(Mean±SD)	(Mean±SD)	df	t	p
LJT							
	Right	Dorsal	912.8±147.4	914.1±155.7	26	0.05	.520
		Palmar	1098.5±285.6	913.5±180.3	26	4.74	< .001
	Left	Dorsal	901.8±157.6	992.1±297.9	26	2.58	.992
		Palmar	1093.9±285.1	974.0±226.7	26	3.65	< .001
SDJT							
	Right	Dorsal	1080.4±230.3	1034.9±187.9	26	1.46	.079
		Palmar	1114.2±275.9	1106.4±234.9	26	0.28	.393
	Left	Dorsal	1091.7±186.1	1133.6±227.2	26	1.93	.968
		Palmar	1071.6±298.8	1081.9±234.5	26	0.45	.630

Note. Each mean RT and its SD was calculated from RTs at 45°, 90° and 135° in each rotation direction. LJT = Laterality Judgment Task. SDJT = Same-Different Judgment Task. SD = standard deviation. df = degree of freedom

Supplementary table 4. Results of between-view comparisons (significance level in the individual level analyses: $p < .05$)

Task	Hand	Participants who showed	Dorsal	Palmar	$\chi 2$	p*
LJT	Right	Biomechanical constraints effect	6	18	9.00	.003
		Linear angle-RT relationship	25	14	11.0	< .001
		Both of them	6	8	0.33	.564
		None of them	2	3	0.33	.560
	Left	Biomechanical constraints effect	1	12	9.31	.002
		Linear angle-RT relationship	24	25	0.20	.655
		Both of them	1	7	4.50	.034
		None of them	3	2	0.20	.655
SDJT	Right	Biomechanical constraints effect	6	6	0.00	1.00
		Linear angle-RT relationship	26	26	0.00	1.00
		Both of them	5	6	0.14	.706
		None of them	0	1	1.00	.317
	Left	Biomechanical constraints effect	2	2	0.00	1.00
		Linear angle-RT relationship	26	26	0.00	1.00
		Both of them	2	2	0.00	1.00
		None of them	2	2	0.00	1.00

Note. LJT = Laterality Judgment Task. SDJT = Same-Different Judgment Task. RT = response time. NA = not applicable.

* Eight tests were conducted for each task. We, therefore, the significance level of this comparison was set at $p < 0.05/8 = 0.00625$ (Bonferroni correction).

Supplementary table 5. Results of between-view comparisons (significance level in the individual level analyses: $p < .2$)

Task	Hand	Participants who showed	Dorsal	Palmar	$\chi 2$	p*
LJT	Right	Biomechanical constraints effect	8	19	8.06	.005
		Linear angle-RT relationship	26	19	5.44	.020
		Both of them	8	13	2.27	.132
		None of them	1	2	0.33	.564
	Left	Biomechanical constraints effect	6	17	7.12	.008
		Linear angle-RT relationship	25	20	3.57	.059
		Both of them	6	11	1.92	.166
		None of them	2	1	0.33	.564
SDJT	Right	Biomechanical constraints effect	14	8	4.50	.033
		Linear angle-RT relationship	26	27	1.00	.317
		Both of them	13	8	3.57	.059
		None of them	0	0	NA	NA
	Left	Biomechanical constraints effect	5	5	0.00	1.00
		Linear angle-RT relationship	27	25	2.00	.157
		Both of them	5	5	0.00	1.00
		None of them	0	2	2.00	.157

Note. LJT = Laterality Judgment Task. SDJT = Same-Different Judgment Task. RT = response time. NA = not applicable.

* Eight tests were conducted for each task. We, therefore, the significance level of this comparison was set at $p < 0.05/8 = 0.00625$ (Bonferroni correction).

Supplementary table 6. Results of between-task comparison (siginificance level in the individual level analyses: $p < .05$)

Hand	View	Participants who showed	LJT	SDJT	$\chi 2$	p*
Right	Dorsal	Biomechanical constraints effect	6	6	0.00	1.00
		Linear angle-RT relationship	25	26	0.33	.564
		Both of them	6	5	0.11	.739
		None of them	2	0	2.00	.157
	Palmar	Biomechanical constraints effect	18	6	9.00	.003
		Linear angle-RT relationship	14	26	12.0	< .001
		Both of them	8	6	0.40	.527
		None of them	3	1	1.00	.317
Left	Dorsal	Biomechanical constraints effect	1	2	0.33	.564
		Linear angle-RT relationship	24	25	0.20	.655
		Both of them	1	2	0.33	.564
		None of them	3	2	0.20	.655
	Palmar	Biomechanical constraints effect	12	2	8.33	.004
		Linear angle-RT relationship	20	25	3.57	.059
		Both of them	7	2	3.57	.059
		None of them	2	2	0.00	1.00

Note. RT = resonse time. NA = not applicable.

* Eight tests were conducted for each hand condition. We, therefore, set the significance level at $p < 0.05/8 = 0.00625$ (Bonferroni correction).

Supplementary table 7. Results of between-task comparison (significance level in the individual level analyses: $p < .2$)

Hand	View	Participants who showed	LJT	SDJT	$\chi 2$	p*
Right	Dorsal	Biomechanical constraints effect	8	14	3.00	.083
		Linear angle-RT relationship	26	26	0.00	1.00
		Both of them	8	13	1.92	.166
		None of them	1	0	1.00	.317
	Palmar	Biomechanical constraints effect	19	8	6.37	.012
		Linear angle-RT relationship	19	27	8.00	.005
		Both of them	13	8	1.67	.197
		None of them	2	0	2.00	.157
Left	Dorsal	Biomechanical constraints effect	6	5	0.11	.739
		Linear angle-RT relationship	25	27	2.00	.157
		Both of them	6	5	0.11	.739
		None of them	2	0	2.00	.157
	Palmar	Biomechanical constraints effect	17	5	8.00	.005
		Linear angle-RT relationship	20	25	3.57	.059
		Both of them	11	5	2.57	.109
		None of them	1	2	1.00	.317

Note. RT = resonse time. NA = not applicable.

* Eight tests were conducted for each hand condition. We, therefore, set the significance level at $p < 0.05/8 = 0.00625$ (Bonferroni correction).

Supplementary table 8. Regression analysis for RT profiles at individual level (test for presence of linear angle-RT relationship)

				Coefficient							
	Condition			LJT				SDJT			
Subject	Hand	View	Direction	Beta	SE	t	p	Beta	SE	t	p
3	Right	Dorsal	Lateral	1.79	0.31	5.77	< .001	4.14	1.25	3.30	.002
			Medial	1.70	0.31	5.56	< .001	3.22	0.69	4.70	< .001
		Palmar	Lateral	0.47	0.76	0.62	.541	2.99	0.75	3.96	< .001
			Medial	1.13	0.79	1.43	.158	2.94	0.88	3.34	.002
	Left	Dorsal	Lateral	1.74	0.37	4.67	< .001	2.11	1.12	1.89	.065
			Medial	2.07	0.38	5.38	< .001	2.42	0.88	2.75	.008
		Palmar	Lateral	0.84	0.82	1.03	.308	2.54	0.62	4.08	< .001
			Medial	1.34	0.68	1.98	.053	1.87	0.93	2.00	.051
4	Right	Dorsal	Lateral	1.90	0.27	7.07	< .001	1.81	0.31	5.91	< .001
			Medial	2.30	0.29	7.83	< .001	1.97	0.21	9.31	< .001
		Palmar	Lateral	1.75	0.43	4.04	< .001	2.13	0.38	5.61	< .001
			Medial	1.52	0.55	2.74	.008	1.66	0.27	6.14	< .001
	Left	Dorsal	Lateral	1.93	0.43	4.47	< .001	2.68	0.30	8.90	< .001
			Medial	2.08	0.44	4.70	< .001	3.27	0.40	8.09	< .001
		Palmar	Lateral	2.64	0.57	4.62	< .001	1.88	0.32	5.92	< .001
			Medial	2.85	0.59	4.84	< .001	1.89	0.30	6.40	< .001
6	Right	Dorsal	Lateral	3.02	0.45	6.74	< .001	3.19	0.54	5.92	< .001
			Medial	3.43	0.50	6.92	< .001	3.99	0.63	6.29	< .001
		Palmar	Lateral	0.83	0.44	1.87	.067	2.77	0.40	6.86	< .001
			Medial	0.61	0.48	1.26	.214	1.91	0.36	5.32	< .001
	Left	Dorsal	Lateral	1.17	0.43	2.74	.008	1.64	0.43	3.85	< .001
			Medial	1.04	0.37	2.79	.007	2.80	0.52	5.42	< .001
		Palmar	Lateral	2.34	0.67	3.48	.001	2.47	0.42	5.84	< .001
			Medial	1.79	0.72	2.47	.017	2.09	0.44	4.80	< .001
7	Right	Dorsal	Lateral	3.52	0.71	4.94	< .001	4.11	0.81	5.08	< .001
			Medial	3.49	0.74	4.74	< .001	4.50	0.94	4.77	< .001
		Palmar	Lateral	0.48	0.47	1.02	.312	3.62	0.74	4.88	< .001
			Medial	0.38	0.39	0.97	.334	4.18	0.71	5.85	< .001
	Left	Dorsal	Lateral	3.14	0.59	5.31	< .001	4.59	0.92	4.98	< .001
			Medial	3.31	0.58	5.68	< .001	4.83	0.93	5.17	< .001
		Palmar	Lateral	6.50	0.86	7.55	< .001	5.22	0.69	7.60	< .001
			Medial	5.94	0.92	6.49	< .001	4.93	0.59	8.35	< .001
8	Right	Dorsal	Lateral	2.86	0.60	4.79	< .001	4.28	0.51	8.34	< .001

			Medial	2.98	0.52	5.72	< .001	5.05	0.52	9.74	< .001
		Palmar	Lateral	3.36	0.58	5.79	< .001	3.56	0.33	10.93	< .001
			Medial	3.90	0.57	6.84	< .001	3.60	0.40	8.96	< .001
	Left	Dorsal	Lateral	3.16	0.50	6.30	< .001	5.69	0.57	9.94	< .001
			Medial	3.27	0.48	6.88	< .001	5.22	0.60	8.74	< .001
		Palmar	Lateral	2.34	0.63	3.73	< .001	4.99	0.42	11.79	< .001
			Medial	3.17	0.59	5.42	< .001	4.75	0.43	11.09	< .001
9	Right	Dorsal	Lateral	1.54	0.40	3.90	< .001	2.67	0.30	8.91	< .001
			Medial	1.44	0.39	3.65	< .001	2.52	0.46	5.46	< .001
		Palmar	Lateral	1.40	0.47	2.96	.004	2.24	0.49	4.54	< .001
			Medial	0.42	0.43	0.96	.339	2.24	0.47	4.77	< .001
	Left	Dorsal	Lateral	0.92	0.39	2.33	.023	2.37	0.47	5.04	< .001
			Medial	1.24	0.39	3.17	.002	2.70	0.34	8.00	< .001
		Palmar	Lateral	2.51	0.70	3.58	< .001	3.03	0.63	4.82	< .001
			Medial	2.44	0.73	3.34	.001	3.15	0.60	5.20	< .001
10	Right	Dorsal	Lateral	11.02	1.80	6.13	< .001	4.85	1.46	3.32	.002
			Medial	11.89	1.77	6.71	< .001	5.26	1.10	4.76	< .001
		Palmar	Lateral	1.43	1.52	0.94	.351	4.20	0.96	4.39	< .001
			Medial	2.78	1.44	1.93	.059	3.45	0.76	4.55	< .001
	Left	Dorsal	Lateral	11.65	1.63	7.17	< .001	4.07	0.79	5.15	< .001
			Medial	17.82	2.51	7.09	< .001	5.74	1.07	5.35	< .001
		Palmar	Lateral	4.62	1.53	3.03	.004	2.20	1.85	1.18	.241
			Medial	3.72	1.14	3.28	.002	5.32	1.19	4.47	< .001
11	Right	Dorsal	Lateral	4.38	0.72	6.12	< .001	1.67	0.35	4.77	< .001
			Medial	4.10	0.76	5.42	< .001	1.52	0.30	5.01	< .001
		Palmar	Lateral	2.03	0.67	3.02	.004	4.49	0.69	6.55	< .001
			Medial	2.90	0.76	3.82	< .001	4.31	0.68	6.34	< .001
	Left	Dorsal	Lateral	4.50	0.73	6.21	< .001	2.95	0.51	5.82	< .001
			Medial	5.30	0.77	6.88	< .001	3.72	0.70	5.28	< .001
		Palmar	Lateral	3.29	0.73	4.50	< .001	3.53	0.39	8.96	< .001
			Medial	3.88	0.73	5.34	< .001	3.27	0.41	7.93	< .001
13	Right	Dorsal	Lateral	5.17	0.82	6.32	< .001	4.05	0.56	7.24	< .001
			Medial	6.82	1.01	6.77	< .001	4.21	0.60	7.04	< .001
		Palmar	Lateral	4.69	0.83	5.64	< .001	4.92	0.70	7.02	< .001
			Medial	4.17	0.89	4.67	< .001	4.47	0.73	6.16	< .001
	Left	Dorsal	Lateral	7.55	0.96	7.88	< .001	2.70	0.50	5.37	< .001
			Medial	7.72	0.92	8.40	< .001	3.90	0.63	6.20	< .001
		Palmar	Lateral	5.92	0.84	7.01	< .001	4.31	0.73	5.92	< .001
			Medial	5.03	0.92	5.49	< .001	4.75	0.88	5.38	< .001

16	Right	Dorsal	Lateral	2.82	0.71	3.94	< .001	4.18	0.52	8.05	< .001
			Medial	2.27	0.41	5.54	< .001	4.25	0.55	7.73	< .001
		Palmar	Lateral	1.58	0.96	1.64	.107	4.13	0.63	6.58	< .001
			Medial	1.01	0.70	1.44	.155	3.54	0.49	7.19	< .001
	Left	Dorsal	Lateral	1.84	0.40	4.60	< .001	3.61	0.56	6.49	< .001
			Medial	2.18	0.42	5.16	< .001	3.86	0.60	6.40	< .001
		Palmar	Lateral	2.31	0.65	3.53	< .001	5.58	0.71	7.90	< .001
			Medial	2.34	0.68	3.42	.001	5.56	0.67	8.35	< .001
18	Right	Dorsal	Lateral	8.89	1.98	4.50	< .001	5.52	0.53	10.36	< .001
			Medial	10.24	1.90	5.40	< .001	5.93	0.51	11.69	< .001
		Palmar	Lateral	6.69	1.25	5.38	< .001	8.35	1.27	6.58	< .001
			Medial	6.97	0.99	7.01	< .001	7.31	1.37	5.33	< .001
	Left	Dorsal	Lateral	11.33	1.87	6.07	< .001	6.33	0.97	6.56	< .001
			Medial	13.18	1.91	6.92	< .001	6.78	0.96	7.04	< .001
		Palmar	Lateral	6.89	1.37	5.03	< .001	5.41	0.68	8.00	< .001
			Medial	8.18	1.37	5.97	< .001	5.65	0.53	10.62	< .001
19	Right	Dorsal	Lateral	4.85	0.59	8.16	< .001	3.98	0.53	7.51	< .001
			Medial	4.16	0.59	7.01	< .001	4.66	0.64	7.26	< .001
		Palmar	Lateral	1.87	0.33	5.68	< .001	4.89	0.56	8.77	< .001
			Medial	1.92	0.36	5.38	< .001	4.69	0.56	8.42	< .001
	Left	Dorsal	Lateral	4.99	0.80	6.24	< .001	2.03	0.45	4.55	< .001
			Medial	5.25	0.77	6.86	< .001	2.42	0.46	5.28	< .001
		Palmar	Lateral	2.20	0.60	3.69	< .001	4.18	1.05	3.99	< .001
			Medial	2.41	0.58	4.19	< .001	2.29	0.65	3.55	< .001
21	Right	Dorsal	Lateral	2.65	0.95	2.80	.007	4.91	0.75	6.56	< .001
			Medial	1.23	0.80	1.54	.130	5.10	0.84	6.10	< .001
		Palmar	Lateral	2.10	0.89	2.36	.022	5.99	1.18	5.07	< .001
			Medial	1.53	0.80	1.91	.061	4.64	0.94	4.92	< .001
	Left	Dorsal	Lateral	2.59	0.65	3.98	< .001	3.11	0.68	4.61	< .001
			Medial	2.46	0.97	2.54	.014	3.22	0.48	6.65	< .001
		Palmar	Lateral	1.01	0.74	1.37	.178	2.80	0.73	3.86	< .001
			Medial	0.67	0.69	0.97	.339	2.70	0.64	4.23	< .001
23	Right	Dorsal	Lateral	3.94	0.55	7.11	< .001	2.38	0.48	4.97	< .001
			Medial	4.07	0.57	7.14	< .001	2.80	0.50	5.58	< .001
		Palmar	Lateral	3.25	0.86	3.79	< .001	4.38	0.56	7.79	< .001
			Medial	3.25	0.84	3.86	< .001	3.50	0.55	6.30	< .001
	Left	Dorsal	Lateral	3.96	0.52	7.60	< .001	5.07	0.54	9.38	< .001
			Medial	4.01	0.51	7.91	< .001	4.83	0.52	9.32	< .001
		Palmar	Lateral	2.77	0.62	4.46	< .001	2.78	0.49	5.67	< .001

			Medial	2.57	0.76	3.39	.001	2.88	0.48	5.95	< .001
25	Right	Dorsal	Lateral	5.06	0.61	8.31	< .001	3.17	0.42	7.49	< .001
			Medial	5.61	0.59	9.56	< .001	3.66	0.51	7.21	< .001
		Palmar	Lateral	1.39	0.64	2.17	.034	3.46	0.51	6.73	< .001
			Medial	0.54	0.43	1.25	.218	3.72	0.76	4.88	< .001
	Left	Dorsal	Lateral	4.90	0.75	6.50	< .001	2.49	0.51	4.86	< .001
			Medial	5.05	0.77	6.58	< .001	3.27	0.58	5.66	< .001
		Palmar	Lateral	0.57	0.66	0.87	.387	4.28	0.52	8.28	< .001
			Medial	0.25	0.52	0.47	.638	4.27	0.46	9.25	< .001
26	Right	Dorsal	Lateral	-0.91	0.60	-1.51	.137	3.39	0.57	5.93	< .001
			Medial	-1.64	0.71	-2.31	.025	3.82	0.48	7.99	< .001
		Palmar	Lateral	0.61	0.35	1.76	.084	3.40	0.55	6.18	< .001
			Medial	1.23	0.41	2.97	.005	3.43	0.59	5.84	< .001
	Left	Dorsal	Lateral	0.22	0.60	0.37	.715	3.60	0.63	5.71	< .001
			Medial	-0.10	0.57	-0.17	.866	4.50	0.94	4.77	< .001
		Palmar	Lateral	-0.26	0.69	-0.37	.710	3.25	0.52	6.27	< .001
			Medial	-0.06	0.53	-0.11	.916	3.59	0.62	5.77	< .001
27	Right	Dorsal	Lateral	2.68	0.39	6.89	< .001	4.34	0.77	5.65	< .001
			Medial	2.97	0.38	7.83	< .001	4.15	0.71	5.87	< .001
		Palmar	Lateral	3.80	0.64	5.94	< .001	7.36	1.26	5.82	< .001
			Medial	3.19	0.62	5.18	< .001	6.86	1.23	5.56	< .001
	Left	Dorsal	Lateral	3.32	0.90	3.71	< .001	4.40	0.71	6.21	< .001
			Medial	3.22	0.90	3.59	< .001	6.33	1.05	6.00	< .001
		Palmar	Lateral	5.01	0.61	8.20	< .001	4.62	0.78	5.91	< .001
			Medial	4.35	0.63	6.94	< .001	5.93	0.92	6.48	< .001
28	Right	Dorsal	Lateral	2.83	0.53	5.35	< .001	3.94	0.68	5.83	< .001
			Medial	3.35	0.47	7.12	< .001	4.72	0.68	6.94	< .001
		Palmar	Lateral	0.55	0.62	0.90	.374	3.62	0.68	5.36	< .001
			Medial	0.34	0.43	0.81	.424	2.90	0.70	4.13	< .001
	Left	Dorsal	Lateral	3.13	0.64	4.89	< .001	3.35	0.64	5.20	< .001
			Medial	3.65	0.58	6.32	< .001	4.63	0.68	6.80	< .001
		Palmar	Lateral	0.48	0.74	0.65	.517	5.29	0.95	5.59	< .001
			Medial	-0.10	0.46	-0.23	.819	5.05	0.90	5.61	< .001
29	Right	Dorsal	Lateral	1.48	0.25	5.89	< .001	4.55	0.70	6.47	< .001
			Medial	1.50	0.29	5.18	< .001	4.51	0.70	6.45	< .001
		Palmar	Lateral	1.45	0.28	5.10	< .001	3.17	0.57	5.60	< .001
			Medial	1.05	0.29	3.62	< .001	2.16	0.48	4.53	< .001
	Left	Dorsal	Lateral	0.45	0.23	1.98	.052	1.94	0.33	5.84	< .001
			Medial	0.49	0.20	2.46	.017	2.01	0.42	4.81	< .001

		Palmar	Lateral	2.20	0.36	6.16	< .001	2.32	0.48	4.85	< .001
			Medial	2.26	0.36	6.35	< .001	2.49	0.42	5.94	< .001
32	Right	Dorsal	Lateral	6.59	1.16	5.67	< .001	9.83	1.73	5.69	< .001
			Medial	7.37	1.07	6.86	< .001	9.56	1.80	5.32	< .001
		Palmar	Lateral	5.73	0.93	6.19	< .001	10.31	1.67	6.17	< .001
			Medial	4.28	0.88	4.86	< .001	10.66	2.06	5.17	< .001
	Left	Dorsal	Lateral	1.36	1.50	0.91	.368	6.54	1.31	5.00	< .001
			Medial	2.74	1.53	1.79	.079	8.58	1.54	5.56	< .001
		Palmar	Lateral	7.80	1.03	7.56	< .001	8.17	1.24	6.60	< .001
33			Medial	6.37	1.05	6.07	< .001	9.30	1.26	7.40	< .001
	Right	Dorsal	Lateral	3.12	0.66	4.70	< .001	8.17	1.01	8.07	< .001
			Medial	3.84	0.59	6.53	< .001	6.57	1.11	5.93	< .001
		Palmar	Lateral	0.92	0.48	1.91	.061	8.48	1.53	5.55	< .001
			Medial	1.72	0.75	2.29	.026	8.23	1.03	7.97	< .001
	Left	Dorsal	Lateral	2.59	0.34	7.65	< .001	3.50	1.23	2.84	.006
			Medial	3.21	0.43	7.43	< .001	2.63	1.36	1.93	.058
		Palmar	Lateral	2.08	0.49	4.26	< .001	8.02	1.71	4.70	< .001
			Medial	2.80	0.50	5.58	< .001	6.13	1.68	3.64	< .001
34	Right	Dorsal	Lateral	2.76	0.60	4.56	< .001	2.57	0.39	6.63	< .001
			Medial	3.54	0.72	4.91	< .001	2.81	0.32	8.86	< .001
		Palmar	Lateral	1.81	0.53	3.45	.001	3.97	0.42	9.57	< .001
			Medial	1.65	0.55	3.02	.004	3.87	0.49	7.94	< .001
	Left	Dorsal	Lateral	3.54	0.58	6.07	< .001	3.16	0.47	6.67	< .001
			Medial	3.20	0.63	5.04	< .001	4.17	0.48	8.71	< .001
		Palmar	Lateral	1.91	0.36	5.33	< .001	3.06	0.40	7.66	< .001
			Medial	1.53	0.35	4.36	< .001	2.72	0.42	6.47	< .001
35	Right	Dorsal	Lateral	4.46	0.65	6.84	< .001	3.13	0.43	7.25	< .001
			Medial	4.30	0.67	6.46	< .001	2.99	0.43	6.91	< .001
		Palmar	Lateral	2.40	0.58	4.14	< .001	2.46	0.26	9.36	< .001
			Medial	2.47	0.53	4.63	< .001	2.37	0.29	8.05	< .001
	Left	Dorsal	Lateral	4.44	0.77	5.77	< .001	1.98	0.36	5.42	< .001
			Medial	4.61	0.73	6.36	< .001	1.78	0.33	5.36	< .001
		Palmar	Lateral	3.89	0.76	5.09	< .001	2.72	0.37	7.26	< .001
			Medial	3.96	0.71	5.55	< .001	3.18	0.48	6.69	< .001
36	Right	Dorsal	Lateral	4.45	0.81	5.51	< .001	1.96	0.44	4.43	< .001
			Medial	5.87	0.91	6.44	< .001	2.34	0.39	6.07	< .001
		Palmar	Lateral	4.06	1.73	2.34	.024	1.98	0.39	5.10	< .001
			Medial	2.26	0.86	2.63	.011	2.06	0.46	4.44	< .001
	Left	Dorsal	Lateral	3.39	0.46	7.33	< .001	1.58	0.42	3.75	< .001

			Medial	3.91	0.47	8.24	< .001	1.95	0.37	5.24	< .001
		Palmar	Lateral	3.70	1.24	2.98	.005	1.67	0.40	4.20	< .001
			Medial	1.66	0.80	2.06	.044	1.97	0.46	4.27	< .001
37	Right	Dorsal	Lateral	3.52	0.51	6.91	< .001	3.86	0.49	7.83	< .001
			Medial	3.51	0.49	7.24	< .001	4.18	0.54	7.74	< .001
		Palmar	Lateral	1.93	0.98	1.96	.055	4.02	0.52	7.71	< .001
			Medial	1.53	0.51	3.00	.004	4.04	0.61	6.65	< .001
	Left	Dorsal	Lateral	2.15	0.58	3.69	< .001	3.80	0.50	7.60	< .001
			Medial	2.86	0.67	4.25	< .001	4.11	0.69	5.97	< .001
		Palmar	Lateral	-0.42	0.85	-0.49	.624	3.79	0.52	7.22	< .001
			Medial	0.32	0.82	0.40	.694	4.38	0.63	6.99	< .001
38	Right	Dorsal	Lateral	1.40	0.37	3.84	< .001	2.04	0.30	6.84	< .001
			Medial	1.68	0.34	4.90	< .001	2.06	0.32	6.53	< .001
		Palmar	Lateral	1.78	0.62	2.89	.007	2.90	0.30	9.69	< .001
			Medial	1.02	0.48	2.15	.038	2.90	0.29	9.86	< .001
	Left	Dorsal	Lateral	1.71	0.32	5.27	< .001	2.66	0.45	5.96	< .001
			Medial	1.57	0.34	4.65	< .001	2.98	0.45	6.71	< .001
		Palmar	Lateral	0.59	0.49	1.20	.236	3.41	0.52	6.52	< .001
			Medial	0.21	0.40	0.53	.600	3.34	0.56	5.97	< .001
39	Right	Dorsal	Lateral	2.60	0.58	4.51	< .001	0.77	0.81	0.95	.348
			Medial	2.97	0.53	5.60	< .001	0.98	0.69	1.41	.163
		Palmar	Lateral	0.82	0.65	1.25	.215	3.22	0.89	3.60	< .001
			Medial	0.56	0.46	1.21	.234	1.23	0.77	1.59	.118
	Left	Dorsal	Lateral	2.67	0.55	4.85	< .001	4.18	0.75	5.60	< .001
			Medial	2.82	0.57	4.96	< .001	4.03	0.78	5.19	< .001
		Palmar	Lateral	2.19	0.56	3.90	< .001	2.14	0.77	2.76	.008
			Medial	2.56	0.55	4.68	< .001	2.43	0.70	3.46	.001

Note. LJT = Laterality Judgment Task. SDJT = Same-Different Judgment Task. SD = standard deviation. df = degree of freedom.

Supplementary table 9. Comparisons of RTs between medial and lateral rotation at individual lelvel (test for presence of biomechanical constraints effect)

			LJT					SDJT				
	Condition		Response time (Mean±SD)					Response time (Mean±SD)				
Subject	Hand	View	Lateral	Medial	df	t	p	Lateral	Medial	df	t	p
3	Right	Dorsal	790.1±120.6	721.1±76.7	64	2.79	.004	1703.9±681.1	1307.1± 356.0	61	2.98	.002
		Palmar	1079.7±359.5	1017.8±387.4	67	0.69	.247	1367.6±437.1	1251.4± 524.8	65	0.99	.163
	Left	Dorsal	920.8±194.6	955.4±223.4	66	0.68	.751	1415.7±540.3	1316.3±353.2	66	0.89	.188
		Palmar	1179.5±417.2	1186.5±313.4	66	0.08	.531	1292.0±296.2	1328.0±487.6	66	0.37	.643
4	Right	Dorsal	722.4±109.3	733.3±176.7	61	0.30	.616	893.9±177.5	839.8±138.5	66	1.40	.083
		Palmar	718.6±147.4	796.1±256.8	67	1.54	.936	868.9±242.3	854.9±144.8	66	0.29	.387
	Left	Dorsal	738.7±119.2	716.9±131.7	67	0.72	.237	877.6±160.7	959.3±283.1	64	1.43	.921
		Palmar	681.0±153.8	658.9±176.5	67	0.55	.290	790.0±148.0	740.7±112.2	67	1.56	.062
6	Right	Dorsal	862.6±151.5	897.2±255.4	60	0.64	.737	955.1±228.1	1071.6±338.5	64	1.64	.947
		Palmar	716.1±123.3	723.6±162.1	53	0.64	.021	891.6±255.8	927.1±150.3	65	0.69	.754
	Left	Dorsal	949.6±225.0	856.1±154.7	64	1.96	.027	981.9±215.5	988.5±332.6	60	0.09	.537
		Palmar	1007.2±321.3	1013.6±332.2	66	0.08	.532	870.0±166.4	870.4±166.4	65	0.01	.504
7	Right	Dorsal	1083.0±271.7	1045.3±281.3	66	0.56	.288	1156.8±231.9	1238.4±435.2	64	0.95	.828
		Palmar	1047.3±260.5	927.6±207.4	66	2.10	.020	1105.9±283.8	1201.9±328.4	64	1.27	.895
	Left	Dorsal	1086.9±242.3	1299.4±307.6	63	3.09	.999	1294.3±371.8	1235.7±400.0	67	0.63	.266
		Palmar	1214.2±326.9	996.2±245.0	66	1.42	.081	1126.4±376.2	1160.1±282.1	65	0.41	.660
8	Right	Dorsal	1003.4±274.7	1015.6±225.5	64	0.20	.578	957.5±195.0	1046.7±326.3	62	1.32	.904
		Palmar	905.9±215.2	909.0±250.6	62	0.05	.521	944.6±187.7	1053.0±232.3	65	2.10	.980
	Left	Dorsal	979.0±179.2	924.9±161.1	60	1.25	.108	1213.8±329.3	1009.9±221.2	63	2.92	.002
		Palmar	885.7±169.0	919.4±221.1	65	0.70	.757	974.5±226.0	917.4±173.9	65	1.16	.125
9	Right	Dorsal	705.8±196.2	686.1±14t3.5	65	0.46	.322	916.5±192.7	901.2±281.4	67	0.26	.397
		Palmar	842.8±152.9	637.5±107.3	63	4.27	< .001	996.2±288.3	969.9±275.1	66	0.39	.351
	Left	Dorsal	668.6±119.2	710.5±152.9	63	1.23	.112	939.3±282.5	882.5±218.8	65	0.92	.181
		Palmar	768.8±178.7	763.7±189.6	65	0.11	.455	902.0±246.9	903.8±215.7	66	0.03	.513
10	Right	Dorsal	1105.7±426.3	1124.7±483.9	64	0.17	.567	1675.0±677.8	1209.9±381.7	64	3.41	< .001
		Palmar	1668.4±628.4	1113.7±397.3	65	4.30	< .001	1477.5±531.2	1274.3±368.2	65	1.82	.036
	Left	Dorsal	1366.4±555.1	2210.6±1859. 8	66	2.50	.993	1302.7±371.9	1530.6±648.1	65	1.74	.957
		Palmar	2013.7±851.8	1557.4±619.8	68	2.56	.006	1629.2±1047.0	1587.3±723.1	64	0.19	.426
11	Right	Dorsal	866.4±237.0	853.3±261.7	67	0.22	.414	962.2±206.4	908.0±152.7	64	1.21	.115
		Palmar	1155.9±328.1	1177.7±431.3	66	0.23	.591	1104.8±261.8	987.0±176.0	65	2.16	.017
	Left	Dorsal	999.2±209.5	1021.1±388.8	66	0.29	.614	995.1±190.5	1110.7±414.3	64	1.46	.925
		Palmar	1263.2±262.0	1131.7±287.8	65	1.95	.028	965.3±216.2	938.1±192.1	66	0.55	.297
13	Right	Dorsal	893.3±232.6	1144.2±612.1	62	2.21	.985	1069.9±276.0	974.8±305.2	63	1.32	.096

		Palmar	1028.4±300.4	868.3±189.9	64	2.57	.006	1152.5±317.6	1023.0±246.2	58	1.76	.042
	Left	Dorsal	842.8±253.0	1001.7±347.2	64	2.14	.982	1076.4±189.5	1186.4±379.9	61	1.42	.919
		Palmar	986.4±352.0	826.0±207.7	66	2.27	.013	1002.9±318.0	1146.9±457.9	63	1.48	.929
16	Right	Dorsal	1206.3±430.7	861.6±155.6	64	4.32	< .001	1079.1±253.2	981.4±263.8	65	1.55	.063
		Palmar	1422.4±418.5	977.0±268.3	66	5.26	< .001	1174.9±368.0	1031.8±222.4	67	1.95	.028
	Left	Dorsal	965.0±197.8	881.3±211.0	63	1.65	.052	1016.6±246.5	1212.2±329.4	64	2.73	.996
		Palmar	1166.5±320.2	1010.6±270.6	66	2.17	.017	931.2±193.5	1036.0±220.3	62	2.02	.976
18	Right	Dorsal	954.9±264.3	1125.6±482.7	64	1.78	.960	1118.0±270.4	1012.5±290.6	65	1.54	.067
		Palmar	1264.7±711.4	1286.9±588.7	64	0.14	.555	1072.1±343.6	1179.0±391.8	65	1.19	.880
	Left	Dorsal	1082.9±317.9	1400.3±826.5	64	2.08	.979	1285.7±385.4	1234.3±409.2	64	0.52	.301
		Palmar	1421.2±787.6	1674.8±874.4	63	1.23	.888	1093.7±413.2	1142.9±364.8	68	0.53	.700
19	Right	Dorsal	1013.1±382.3	784.0±237.0	62	2.84	.003	894.5±253.5	982.7±399.1	65	1.08	.857
		Palmar	729.8±206.0	712.0±226.4	65	0.34	.368	946.5±248.3	887.5±187.5	66	1.10	.138
	Left	Dorsal	827.2±140.0	947.3±245.5	66	2.48	.992	853.2±232.8	895.2±264.2	65	0.69	.754
		Palmar	847.6±158.5	826.0±125.0	66	0.62	.268	1121.8±677.3	962.3±356.4	66	1.20	.116
21	Right	Dorsal	971.1±410.5	831.3±245.1	62	1.65	.052	1184.2±254.0	1249.4±374.9	64	0.82	.793
		Palmar	944.3±394.2	677.3±155.0	64	3.62	< .001	1324.1±673.3	1325.0±473.3	64	0.01	.502
	Left	Dorsal	877.9±255.2	993.1±516.5	62	1.13	.869	1137.7±396.6	1116.3±298.7	64	0.25	.402
		Palmar	1067.6±366.4	931.5±329.5	62	1.56	.061	1335.3±329.7	1124.7±285.8	65	2.79	.004
23	Right	Dorsal	846.9±163.2	834.3±182.3	66	0.30	.383	973.8±265.9	1063.6±303.7	66	1.29	.900
		Palmar	1073.7±305.9	915.1±219.5	61	2.36	.011	1049.3±321.3	1043.9±226.6	63	0.08	.469
	Left	Dorsal	754.9±110.1	809.1±151.6	65	1.67	.950	1093.6±325.5	1022.8±260.8	64	0.97	.167
		Palmar	981.91±198.4	861.9±254.2	61	2.08	.021	898.3±162.6	976.8±198.6	66	1.78	.960
25	Right	Dorsal	1035.9±224.0	1145.9±328.3	65	1.59	.941	877.8±208.0	954.9±301.4	66	1.23	.888
		Palmar	1438.9±349.6	1137.0±225.4	68	4.29	< .001	997.8±300.3	1134.6±476.0	65	1.40	.917
	Left	Dorsal	961.8±188.6	919.8±208.6	67	0.88	.192	981.6±303.8	986.1±387.8	66	0.05	.521
		Palmar	1253.2±313.8	1137.2±191.1	69	1.89	.032	908.8±268.0	903.4±223.2	66	0.09	.464
26	Right	Dorsal	807.4±185.4	1064.7±327.2	61	3.79	.999	924.1±269.0	882.3±230.7	66	0.69	.247
		Palmar	943.0±145.6	814.3±198.3	59	2.91	.003	886.5±286.7	1004.0±316.2	64	1.58	.941
	Left	Dorsal	837.4±252.8	958.3±239.5	65	2.01	.976	951.6±314.8	1108.6±568.2	67	1.41	.919
		Palmar	1159.7±303.2	822.9±213.1	64	5.22	< .001	913.0±294.9	915.9±370.4	65	0.04	.514
27	Right	Dorsal	762.8±145.4	738.7±167.4	63	0.62	.269	1040.6±344.3	923.9±237.5	65	1.61	.056
		Palmar	873.7±317.7	759.6±185.3	64	1.77	.041	1078.4±388.9	1016.7±336.9	61	0.67	.251
	Left	Dorsal	750.9±128.1	819.1±205.3	65	1.63	.946	1022.7±201.5	1261.7±618.0	65	2.01	.980
		Palmar	778.8±263.5	854.0±232.0	64	1.23	.889	1051.6±298.34	1142.9±503.7	66	0.91	.817
28	Right	Dorsal	910.8±228.6	972.4±230.8	62	1.07	.857	1204.0±363.0	1403.6±417.4	64	2.07	.978
		Palmar	1351.3±275.7	1078.6±203.3	65	4.60	< .001	1318.6±428.8	1314.0±401.5	65	0.05	.482
	Left	Dorsal	871.3±191.4	962.5±246.8	65	1.69	.952	1300.7±332.6	1309.7±448.7	64	0.09	.537
		Palmar	1344.4±360.8	1021.8±219.2	66	4.42	< .001	1419.1±553.8	1359.4±508.9	66	0.46	.322

29	Right	Dorsal	676.5±96.1	740.4±145.5	66	2.14	.982	938.8±278.1	875.4±239.0	63	0.98	.164
		Palmar	752.5±143.6	663.0±108.3	66	2.90	.003	925.4±298.7	825.0±146.1	66	1.74	.043
	Left	Dorsal	732.5±113.9	746.3±96.5	66	0.54	.705	839.8±164.7	862.3±228.2	68	0.47	.682
		Palmar	840.3±151.9	821.9±149.5	68	0.51	.306	866.3±213.1	859.1±188.3	61	0.14	.444
32	Right	Dorsal	918.7±276.6	1120.2±4t10.9	58	2.23	.985	1205.8±352.0	1456.1±495.9	64	2.36	.989
		Palmar	1519.7±530.7	1018.7±306.3	66	4.69	< .001	1318.4±489.6	1737.2±978.2	67	2.24	.986
	Left	Dorsal	1085.9±311.2	1290.9±527.6	63	1.92	.970	1405.5±443.7	1608.6±830.1	68	1.27	.895
		Palmar	1174.8±485.5	1017.6±278.8	64	1.64	.053	1343.6±435.2	1423.5±607.4	65	0.62	.731
33	Right	Dorsal	729.0±119.5	835.9±199.4	62	2.60	.994	1553.1±482.6	1308.2±344.0	62	2.35	.011
		Palmar	1033.0±280.0	1129.4±123.2	66	1.15	.873	2131.1±955.4	1708.2±636.9	64	2.14	.018
	Left	Dorsal	694.5±123.2	843.0±288.5	69	2.91	.998	1448.2±494.0	1663.0±581.0	67	1.65	.948
		Palmar	935.7±193.1	795.4±253.7	64	2.53	.007	2140.3±900.5	1742.1±816.8	64	1.88	.032
34	Right	Dorsal	772.2±135.9	852.9±341.5	66	1.27	.895	859.1±197.0	752.4±169.1	60	2.30	.013
		Palmar	827.3±167.0	746.4±145.7	62	2.06	.022	878.5±209.0	903.9±243.6	65	0.46	.677
	Left	Dorsal	873.2±213.6	793.9±175.2	64	1.64	.053	912.2±176.7	899.9±290.9	64	0.21	.419
		Palmar	758.9±172.1	703.7±122.8	64	1.50	.069	813.4±247.9	882.4±234.2	59	1.11	.865
35	Right	Dorsal	1111. 0±258.4	1019.8±198.3	64	1.61	.056	1025.6±222.3	846.0±161.5	66	3.79	< .001
		Palmar	1024.2±257.5	976.4±199.3	66	0.85	.198	927.6±156.9	923.2±166.4	66	0.11	.456
	Left	Dorsal	975.2±176.3	1087.8±262.9	67	2.07	.979	978.2±215.8	955.4±180.3	65	0.47	.320
		Palmar	1097.0±295.0	1018.6±221.2	67	1.25	.107	951.7±187.4	1022.7±298.9	66	1.17	.877
36	Right	Dorsal	997.1±288.7	1104.5±524.3	61	1.02	.844	888.8±209.7	869.3±190.9	63	0.39	.349
		Palmar	1751.5±937.4	1146.8±361.9	58	3.38	< .001	808.2±183.0	918.0±245.9	61	2.01	.975
	Left	Dorsal	807.6±166.3	941.3±291.1	66	2.32	.988	879.5±204.3	826.4±188.7	67	1.12	.133
		Palmar	1550.3±702.1	950.4±284.2	60	4.56	< .001	793.8±194.5	882.4±243.1	61	1.60	.943
37	Right	Dorsal	1035.4±301.0	867.6±255.9	62	2.41	.010	1027.1±264.0	976.9±317.5	64	0.70	.243
		Palmar	1314.2±513.7	747.8±168.9	64	6.02	< .001	1041.3±262.6	1118.1±323.3	63	1.04	.850
	Left	Dorsal	1086.0±254.6	1042.2±354.4	65	0.58	.281	1061.1±270.5	1207.7±400.1	64	1.74	.957
		Palmar	1265.8±428.6	981.8±371.6	63	2.86	.003	900.9±211.6	1043.0±340.9	60	1.99	.974
38	Right	Dorsal	742.7±139.8	663.1±95.3	67	2.76	.004	907.1±166.4	858.9±181.9	62	1.10	.137
		Palmar	1114.0±271.3	815.9±162.1	43	4.54	< .001	858.0±178.9	872.6±178.0	63	0.33	.629
	Left	Dorsal	745.6±185.2	740.3±185.1	66	0.12	.454	964.4±194.9	874.6±213.6	67	1.82	.036
		Palmar	978.2±247.5	877.5±189.9	57	1.76	.042	862.3±172.7	1001.7±190.8	67	3.19	.999
39	Right	Dorsal	1078.4±267.4	916.8±238.2	63	2.58	.006	1162.7±367.0	979.4±177.8	65	2.59	.006
		Palmar	1095.0±330.8	873.8±181.5	63	3.35	< .001	1402.8±484.7	1359.1±357.9	66	0.43	.336
	Left	Dorsal	815.7±232.0	900.5±285.7	64	1.32	.904	1261.2±399.2	1300.7±406.9	66	0.40	.656
		Palmar	834.8±168.2	846.4±177.5	64	0.27	.607	1022.7±260.5	1163.7±252.2	68	2.31	.988

Note. LJT = Laterality Judgment Task. SDJT = Same-Different Judgment Task. SD = standard deviation. df = degree of freedom.
